# Cultural similarity predicts social inclusion of Muslims in Canada: A vignette-based experimental survey

**DOI:** 10.3389/fpsyg.2022.973603

**Published:** 2022-11-22

**Authors:** Hajra Tahir, Saba Safdar

**Affiliations:** ^1^Faculty of Psychology, University of Bergen, Bergen, Norway; ^2^Department of Psychology, University of Guelph, Guelph, ON, Canada

**Keywords:** acculturation, intergroup emotions theory, social exclusion, Canada, Muslims

## Abstract

Based on acculturation psychology and intergroup emotions theory, the current experimental study assessed the effects of Muslims’ perceived acculturation strategies by the majority group on social exclusion of Muslims in Canada, and to what extent religious resentment mediated the relationship between Muslims’ perceived acculturation strategies and social exclusion. The experimental study used a vignette-based approach. This model was examined among 190 non-Muslim Canadians. Results showed that when Muslims were viewed as assimilated in Canadian society, social exclusion of Muslims and religious resentment toward Muslims decreased. Furthermore, religious resentment mediated the association between Muslims’ perceived acculturation strategies and social exclusion only when Muslims were perceived as assimilated. Our findings suggest that Canadian majority-group members indicated positive attitude toward Muslims when they were identified as assimilated in Canadian society. Results are discussed in terms of implications for future studies and intergroup relations.

## Introduction

Continuous immigration from Muslim majority countries has changed the religious landscape of Canadian society. In the aftermath of September 11, 2001, the majority group members’ attitudes toward this new transformation have increasingly taken the shape of anti-Muslim sentiments (Yogasingam, 2017). The Islamic belief system, culture, and values are viewed as inconsistent with western norms (Litchmore and Safdar, 2015), while the religious affiliation and adherence of Muslim minorities is considered as a barrier in their societal integration (Foner and Alba, 2008). Based on their religious affiliation, political and public discussions have continuously focused on the cultural orientation of Muslims, i.e., whether Muslims prefer to integrate, assimilate, marginalize, or separate in their respective societies, which in academic research are referred as acculturation strategies (Berry, 1997). For Muslim minorities, their religious identity is an important element of their self-concept, a source of meaning-making, and cultural continuity (Ysseldyk et al., 2010). Research points out that even though most Muslims living in the West prefer to integrate or assimilate (e.g., Sam et al., 2016), the association of Muslims with terrorism and extremism in media and public debates, together with violent crimes orchestrated by radicalized Muslims in western societies have given rise to feelings of resentment toward the Muslim community because this association has increasingly generated views that the Muslim community prefers separation as acculturation strategy (Strelan and Lawani, 2010). Thus, anti-Muslim sentiments and rhetoric in western societies have been closely associated with the social exclusion of the Muslim community from the majority society (Yogasingam, 2017).

Against this backdrop, the present experimental study draws on theories of acculturation (Berry, 1997) and intergroup emotions (Smith, 1993), and attempts to examine the effects of Muslims’ acculturation strategies as perceived by majority group members on the social exclusion of Muslims explained by religious resentment. In doing so, this study aims to contribute to the existing literature on the association between acculturation strategies and intergroup relations.

### Negative out-group behavior due to unsimilar acculturation strategies

Acculturation is defined as a cultural and psychological change and adaptation over time when individuals from different backgrounds come into firsthand contact with each other (Berry, 1997). The four-fold model of acculturation proposed by Berry (1997) establishes four strategies undertaken by individuals in their new society of living. *Integration* occurs when individuals are willing to adopt the culture of the majority society and at the same time, they maintain their cultural heritage. *Assimilation* is the adaption of the majority society’s culture while abandoning heritage culture. *Separation* refers to the preservation of cultural heritage and rejection of the dominant society’s culture. Finally, the *marginalization* category applies to individuals who reject both the majority society and heritage cultures. Several studies have shown that integration and assimilation are associated with the best social and psychological adaptation of minority members (Zagefka and Brown, 2002; Jasinskaja-Lahti et al., 2003; Nguyen and Benet-Martínez, 2013). However, numerous studies have demonstrated that majority group members tend to favor minority group members who share similar values, attitudes, and cultural similarities as them, yielding positive attitudes and behavioral outcomes, such as social inclusion, from majority group members (Bloom et al., 2015). This preference for similarity is also evident in many studies which indicate that majority group members prefer assimilation for immigrants, while they believe that immigrants prefer to maintain their heritage culture only, or choose separation as an acculturation strategy (see e.g., van Oudenhoven and Eisses, 1998; Verkuyten and Thijs, 2002; Florack et al., 2003; Jasinskaja-Lahti et al., 2003; Pfafferot and Brown, 2006; van Oudenhoven et al., 2006; Safdar et al., 2017; Safdar and van de Vijver, 2019). Indeed, several studies have revealed that when majority group members perceive minorities as preferring intergroup contact as an acculturation strategy, they show positive attitudes and behavior toward them (Zagefka et al., 2009; Matera et al., 2011, 2012, 2015). Furthermore, research suggests that minorities’ perceived preference for assimilation may reduce perceptions of intergroup emotions in the form of resentment and dislike among majority group members, which may arise as a negative outcome of intergroup relations (Stephan et al., 1999).

In addition, individuals tend to maintain a positive affiliation with their own group by perceiving their group membership positively. Thus, minority group members who choose to assimilate are perceived as respectful and non-threatening by majority group members because the discrepancy between the acculturation strategy preferred by the majority group members for the minority group, and the acculturation strategy adopted by the minority group is low (see for e.g., Grigoryev and van de Vijver, 2018). On the other hand, minority group members who do not share cultural values with the majority society are viewed as dissimilar due to discord of acculturation strategies, generating unfavorable attitudes and treatment, such as social exclusion, from majority group members (Safdar et al., 2008; Kunst and Sam, 2014).

### Resentment and intergroup behavior

Intergroup emotions theory (IET) developed by Smith (1993) focuses on the role of emotions in intergroup behavior. The theory explains that when people identify with an important social group, group membership becomes an important part of the psychological self and the group attains emotional significance for these individuals. Consequently, a minority group is evaluated according to the social significance they have for the majority group, generating certain intergroup emotions (Seger et al., 2016). Numerous studies have established the direct relationship between emotions and biased intergroup behavior (Stangor et al., 1991; Talaska et al., 2008). Existing literature has also demonstrated that emotions indirectly affect the link between acculturation preferences and negative behavioral tendencies toward minorities, specifically among majority group members, while a few examined the mediating role of emotions on the relationship between acculturation preferences and biased behavior from a majority and minority perspective (Smith and Ellsworth, 1985; Mackie et al., 2000; Zick et al., 2001; Zagefka et al., 2014; Lopez-Rodriguez and Navas, 2016; Olsson et al., 2019). Studies have shown that resentment, as an intergroup emotion, is associated with unfavorable views of minorities within a society (Kinder, 2012). Literature also points out that individuals that are resentful toward minorities tend to perceive high cultural discrepancy and associate the discordance of acculturation attitudes with social inequality (Henry and Sears, 2002). Indeed, high levels of resentment are associated with out-group blaming and negative action tendencies such as violence and social exclusion of minorities (Sieckelinck et al., 2019).

Studies indicate that Islam is viewed as a terroristic threat in European societies (Doosje et al., 2009). Muslims’ religious affiliation and their presence are linked with a higher prevalence of terrorist attacks (Hellevik, 2020), which may give rise to resentment among majority group members toward the Muslim minority. Several studies have supported this association. Bakker-Simonsen and Bonikowski (2020) revealed in their study that Muslims’ religious affiliation was linked with social exclusion in 41 European countries due to feelings of anger and dislike toward Muslims’ religiosity. In the United Kingdom., a study by Helbling and Traunmuller (2020) showed that the majority group members’ negative views toward Muslims were the result of a rejection of Muslims’ religious behavior, which was perceived as a danger to national security and considered as unfit with the democratic values of the British society. A German study by Wallrich et al. (2020) concludes that negative sentiments toward Muslims in Germany were stronger amongst majority group members than negative views against immigrants in general due to their religious behavior.

In this paper, we argue that the Canadian majority group may exhibit religious resentment toward the Muslim community. Even though Islamic inspired terrorism threat is low in Canada compared to the United States, research shows that the majority of Canadians view homegrown Islamic terrorism as a major threat to society (Angus Reid Institute, 2014), and public debates cement Islamic values and Canadian values to be incompatible (Campana and Tanner, 2019). In addition, as Canada shares political, economic, and social ties with the United States, terrorist attacks conducted by radicalized members of the Muslim community in the U. S may have evoked emotional reactions among Canadian majority group members. These experiences of negative emotions may foster resentment toward Muslims’ religious practices in majority group members resulting in negative action tendencies such as the social exclusion of Muslims. In this study, we examined resentment toward Muslims based on their religious affiliation. Religious resentment refers to the degree to which majority group members indicate feelings of anger and dislike toward Muslims due to their religious affiliation. We choose to focus particularly on feelings of resentment toward Muslims and their religious affiliation because studies show that negative out-group behavior is predicted by high levels of resentment and anger (see Mackie et al., 2000). Therefore, since we expected majority group members to socially exclude Muslims due to their religious behavior (Barlow et al., 2019), we examined the effects of resentment as a mediator on the perceived religious acculturation of Muslims in this experimental study.

### Muslims in Canada

Canadian Muslims consist of 3.2% of Canada’s total population and belong to diverse ethnic groups (Statistics Canada, 2016). Officially, Canada became a multicultural country in 1971 when the government adopted a multiculturalism policy through the multicultural act of 1988 (Wood and Gilbert, 2005), which refers to recognizing and valuing political pluralism, and the coexistence of a diverse society as a part of the Canadian identity. However, even though the notion of multiculturalism has been an internationally admirable characteristic of Canadian society, the events of 9/11 challenged this idea intensifying anti-Muslim hate crimes, surveillance of the Muslim community, ethnic profiling by law enforcement, discrimination at jobs, and restrictions on travel within and outside of Canada. As neighbors that share geographical borders, Canada has longstanding economic, political, and social ties with the United States. That is why the terrorist attacks of September 11, San Bernardino in 2015, and the Orlando nightclub shooting in 2016, to name a few, occurred on American soil, impacted Canada, as well as the rest of the world. Consequently, public perceptions of Muslims as law-abiding citizens were replaced with Muslims as intolerant and violent terrorists. In addition, despite being successfully integrated and represented in Canadian society in the media, and in various public and private institutions, Muslims were perceived as resistant to integration, while their lack of social and economic engagement was partly attributed to failed multiculturalism policy (Kazemipur, 2014). Moreover, Canadian Muslims came under intense scrutiny and received increased state attention due to the American Muslim ban and visa restrictions from citizens of Muslim majority countries in 2017 (Elkassem et al., 2018).

### The present research

The purpose of this research is to delve into the Canadian experience with the Muslim minorities with respect to their acculturation strategies as perceived by the majority society and their effects on the social exclusion of Muslims from Canadian society. In a multicultural society, maintaining heritage culture as well as contact with the mainstream society’s culture is not only encouraged, but also linked with positive intergroup relations (Kunst and Sam, 2014). Therefore, the aim of the present study is to examine which acculturation strategies will yield unfavorable outcomes from the majority society. In this way, we tend to explore factors that can contribute to intergroup relations and conflicts, which may influence the relationship between the majority group, and the Muslim minority group in Canada.

Acculturation research commonly focuses on minorities’ heritage culture and the culture of the majority society. In the present research, we examined acculturation in terms of *religious* and the *majority* society’s cultural association because religion is considered an important part of self-identity for Muslims living in western countries (Verkuyten and Yildiz, 2007; Bloom et al., 2015).

Literature suggests that deep-rooted negative attitudes toward an out-group developed through experiences of negative associations are strongly linked with modern, subtle forms of hostile behavior, which may manifest through feelings of resentment toward the out-group (Vollhardt and Bilali, 2015). Muslims in the West have been labeled as barbaric and intolerant than other religious groups, and their religious cultural practices are constructed at odds with western norms and values, predominantly as a consequence of incidents of violence conducted by radicalized members of the Muslim community (Litchmore and Safdar, 2015). Thus, considering these dismaying circumstances we aim to examine attitudes supporting the social exclusion of Muslims by incorporating religious resentment as a mediating factor. The study tends to ascertain the extent to which majority-group members’ feelings of religious resentment toward Muslims mediate the relationship between Muslims’ perceived acculturation strategies and social exclusion endorsed by the majority group.

### Hypotheses

Muslims’ religious values are constantly perceived as inconsistent with western democratic values, whereas research shows that in a European context, majority-group members may show favorable attitudes toward immigrants who prefer to assimilate and integrate into the host society. Public, political, and media debates have negatively framed Muslims’ religious cultural practices and religious acculturation attitudes in Canada in recent years, reflecting the European trend (McCoy et al., 2016). Based on this reasoning, we propose that,

H1: When Muslims are perceived as choosing assimilation and integration as acculturation strategies the participants will not endorse social exclusion of Muslims, whereas when Muslims are perceived as choosing separation and marginalization as acculturation strategies the participants will support social exclusion of Muslims.

Next, feelings of religious resentment toward Muslims in Canada will reflect when Muslims are presented to choose separation and marginalization, but not when Muslims are presented to prefer assimilation or integration as acculturation strategies (Henry and Sears, 2002):

H2: When Muslims are perceived as choosing assimilation and integration as acculturation strategies, the participants will show less religious resentment toward Muslims. Contrarily, when Muslims are perceived as choosing separation and marginalization as acculturation strategies the participants will indicate high levels of religious resentment toward Muslims.

As we expected participants in the assimilation and integration conditions to indicate low levels of religious resentment, while participants in the separation and marginalization conditions are expected to show high levels of religious resentment, we also expect the acculturation strategies to have indirect effects on social exclusion of Muslims mediated by religious resentment.

**FIGURE 1:**
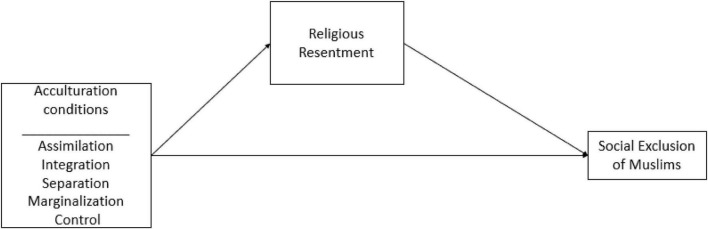
Hypothetical model of the study.

H3: When Muslims are presented as assimilated or integrated in Canadian society, religious resentment toward Muslims will decrease, which in turn is expected to decrease the social exclusion of Muslims. On the contrary, when Muslims are presented as separated and marginalized, religious resentment toward Muslims will increase, which in turn will increase the social exclusion of Muslims (Figure 1).

## Materials and methods

### Participants

An *a priori* power analysis by GPower 3.1 (for a full description, see Faul et al., 2009) with power (1 - β) set at 0.80 and α = 0.05, two-tailed to observe a small effect size (0.02) suggested a sample size of ninety respondents for linear multiple regression analyses for a fixed model with R^2^ deviating from zero. We shared the link to the survey on Amazon Turk, where two hundred and eight Canadians participated. We included only White, non-Muslim participants in the study, and excluded four participants who indicated Islam as their religion. Respondents that resided in Canada and were 18 years and above were included in the study. Ten participants were excluded due to incorrect responses to the attention check item “What is the name of the sport in the text?”. The final sample comprised of 194 participants (Mage = 33.86, SDage = 11.75). Table 1 summarizes the demographic variables in the study.

**TABLE 1:** Demographic variables of the sample (*n* = 190).

Age (*M, SD*)	33.86 (11.75)
Gender women in%	61.9
Employment%	
Unemployed	22.8
Employed	75.1
Retired	2.1
Religious orientation%	
None	27.8
Christianity	34.2
Other	38.0
Education%	
Less than high school	1.2
High school graduate or equivalent	41.9
University	56.0
Income%	
Below Canadian average	48.9
Average	15.4
Above Canadian average	35.6

The remaining missing percentages corresponds to missing responses.

### Procedure

The study received ethical clearance from the University of Guelph’s ethics review board (REB) in Canada prior to data collection. The participants were informed that the study examines immigration and its impact on learning about cultural differences. The survey started with informed consent, which contained information about confidentiality and anonymity. Following the informed consent, the participants completed a demographics section. Next, the participants were randomly allocated to read one of five different texts describing the Muslim community in Canada that prefers assimilation, integration, separation, or marginalization in Canadian society (described below). The fifth text was a control condition. After the text, the participants answered the attention check item. Finally, the participants registered their opinions on the dependent variables; religious resentment, and social exclusion (described below). All scales were measured on a scale ranging from 1 (strongly disagree), to 7 (strongly agree). Analyses were conducted with the PROCESS regression macros (Hayes, 2013). The survey comprised of the following measures.

### Experimental vignettes

Five different vignettes inspired by the Vancouver Index of Acculturation (VIA) developed by Ryder et al. (2000) were used as experimental manipulation and control condition. The texts contained information about Canadian Muslims who either prefer integration, assimilation, separation, or marginalization as acculturation strategies in various life domains, such as values, culture, and entertainment. The fifth text was a control condition. In the assimilation condition, participants read the following text:

Some Muslims strongly care about Canadian cultural heritage only, and not their religious culture. For them, active participation in Canadian cultural traditions, such as dressing up in western clothes, celebrating Christmas, eating hamburgers and apple pie, and beer is very important. They prefer their Canadian culture on their religious culture. It is equally important to them to maintain and develop Canadian cultural practices and values, which is also an important part of their upbringing. They prefer to have friends with non-Muslim, Canadian background and they enjoy social activities with non-Muslim Canadians. They also enjoy entertainment such as Canadian TV shows, sports such as ice hockey, movies, and music. Often their jokes and humor are related to the Canadian culture. Finally, it is very important for them to work with colleagues that belong to the non-Muslim, mainstream society and, therefore, they feel comfortable working with them.

In integration condition, the participants had the following text:

Some Muslims care strongly about their religious and Canadian cultural heritage. For them, active participation in both cultural traditions are very important. It is equally important to them to maintain and develop both culture’s practices and values, which is also an important part of their upbringing. They prefer to have friends with both Muslims and non-Muslim background, and they enjoy social activities with them. They also enjoy entertainment from religious and Canadian culture such as Islamic TV shows, Canadian TV shows, movies, music, and sports such as ice hockey. Often their jokes and humor are related to both cultures. Finally, it is very important for them to work with colleagues that belong to both Muslims and non-Muslim cultures and, therefore, they feel comfortable working with them.

The separation condition contained the following information:

Some Muslims strongly care about their religious cultural heritage only, and not the Canadian culture. For them, active participation in only their religious cultural traditions, such as weekly prayers at the local mosque, dressing up in religious clothes, and fasting during Ramadan is very important. They prefer their religious culture on Canadian culture. It is equally important to them to maintain and develop Islamic cultural practices and values, which is also an important part of their upbringing. They have only Muslim friends and they enjoy social activities with people who are only Muslims. They also enjoy entertainment only from their religious background (such as Islamic TV shows, movies, sports, and music) and do not follow Canadian entertainment or sports such as ice hockey. Often their jokes and humor are related to their religious culture. Finally, it is very important for them to only work with colleagues that share their belief in Islam and, therefore, they feel comfortable working with them.

The text about marginalized Muslims included the following information:

Some Muslims do not care about their religious culture, nor do they prefer to follow the Canadian culture. For them, active participation in both cultural traditions is not important. They do not prefer to maintain and develop either cultures’ practices or values. Neither do they prefer to have friends with either Muslims or non-Muslims. They do not prefer to watch any religious or Canadian entertainment or sport such as ice hockey. Finally, they do not prefer to work with colleagues that belong to either cultures.

The control condition, which did not provide information to the participants about Muslims or any specific acculturation strategy, comprised of the following text:

Some people are known to be vibrant in their community. They actively participate in various traditions, such as dressing up in nice clothes and celebrating festivals, because they consider it very important. For them, it is equally important to maintain and develop these practices and values, because they are an important part of their upbringing. They prefer having friends with different background and they enjoy social activities with others. They also enjoy entertainment such as TV shows, sports such as ice hockey and films. Often their jokes and humour are also witty. They marry by personal choice. Finally, it is very important for them to work and they feel comfortable working with their colleagues.

### Dependent variables

#### Religious resentment toward Muslims

Nine items derived from the Muslim American Resentment scale were adapted from Lajevardi and Kassra (2018) to measure participants’ religious resentment toward Canadian Muslims as a proxy of old and deep-rooted anti-Muslim sentiments (e.g., “Muslims do not have the best interests of Canada at heart”, α = 0.89). Four items were reverse coded.

#### Social exclusion of Muslims

Ten items, with two items reverse coded, specifically designed for the study measured the extent to which the participants were willing to exclude Muslims in various social situations such as at the workplace, renting a portion to Muslim tenants, and voting for a Muslim MP (e.g., “I would not like Muslims as my neighbors”, α = 0.94).

## Result

### Main effects of the conditions on the outcome variables

#### Social exclusion of Muslims

**FIGURE 2:**
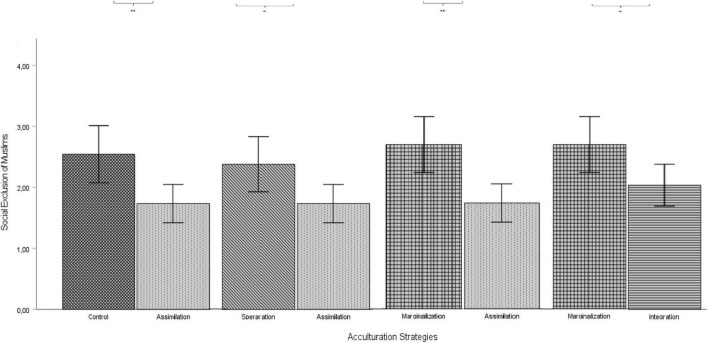
Planned contrasts of various acculturation conditions on social exclusion. Error bars represent 95% confidence intervals.

A one way analysis of variance (ANOVA) indicated a significant omnibus effect of the five conditions on the social exclusion of Muslims, *F*(4, 185) = 3.56, *p* = 0.008, η^2^ = 0.3. Planned contrasts in Figure 2 revealed that social exclusion of Muslims was significantly lower in the perceived assimilation condition than the control group, *t*(185) = -2.79, *p* = 0.006. However, planned contrasts did not reveal any significant effects of Muslims’ perceived integration *t*(185) = -1.76, *p* = 0.08, separation, *t*(185) = -0.57, *p* = 0.57 and marginalization, *t*(185) = 0.51, *p* = 0.61 on social exclusion of Muslims compared to the control group. Hence, H1 was partially confirmed.

Although not part of the hypotheses, we compared the four acculturation strategies with each other, instead of only with the control condition, and examined their effects on the social exclusion of Muslims by using ANOVA. The results of planned contrasts showed that social exclusion of Muslims endorsed by the participants was significantly higher in the separation *t*(185) = 2.23, *p* = 0.027 (Figure 2) and marginalization conditions, *t*(185) = 3.27, *p* = 0.001 compared to the assimilation condition. In addition, social exclusion of Muslims was higher in the marginalization condition compared to the integration condition, *t*(185) = 2.25, *p* = 0.026.

#### Religious resentment toward Muslims

**FIGURE 3:**
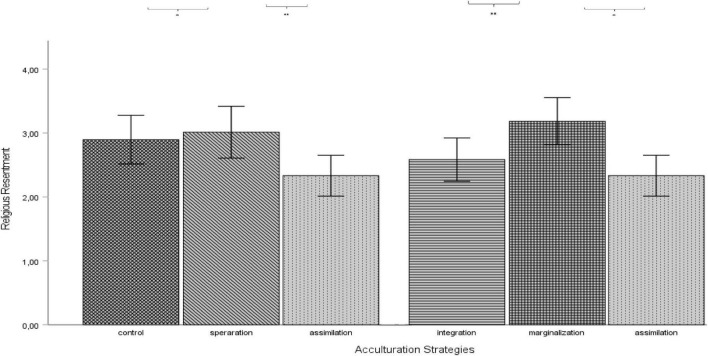
Planned contrasts of various acculturation conditions on religious resentment. Error bars represent 95% confidence intervals.

Results from a one-way ANOVA showed a significant main effect of religious resentment toward Muslims on the various conditions, *F*(4, 185) = 3.55, *p* = 0.008, η^2^ = 0.3. Planned contrasts indicated that religious resentment toward Muslims was lower in the assimilation condition compared to the control group, *t*(185) = -2.22, *p* = 0.028 (Figure 3). However, planned contrasts did not reveal significant effects of integration, *t*(185) = -1.23, *p* = 0.22 separation, *t*(185) = 0.47, *p* = 0.64 and marginalization conditions, *t*(185) = 1.13, *p* = 0.26 on religious resentment, partially confirming H2.

Although not part of the hypotheses, we also compared the four acculturation strategies with each other to examine their main effects on religious resentment toward Muslims. Planned contrasts revealed that religious resentment was significantly higher in the separation *t*(185) = 2.67, *p* = 0.008 (Figure 3) and marginalization conditions, *t*(185) = 3.31, *p* = 0.001 compared to the assimilation condition, whereas religious resentment was higher in the marginalization condition *t*(185) = 2.34, *p* = 0.02 when compared with integration condition.

#### Mediation analysis

Table 2 displays zero order correlations among the independent and dependent variables. We tested the indirect effects of acculturation strategies on social exclusion, mediated by religious resentment. For that purpose, we used Model 4 of PROCESS macro (Hayes, 2013) with bootstrap estimation approach of 5,000 random re-samples for the indirect effect. Unless stated otherwise, the manipulation conditions were coded as integration = 1, assimilation = 2, separation = 3 and marginalization = 4 compared to the control group = 0.

**TABLE 2:** Descriptive statistics and correlations among the main study variables.

**Variable**	**Mean**	**SD**	**1**	**3**
1. Social Exclusion of Muslims	2.28	1.30	1	0.81**
2. Religious Resentment toward Muslims	2.81	1.14	0.81**	1

#### Social exclusion of Muslims

**FIGURE 4:**
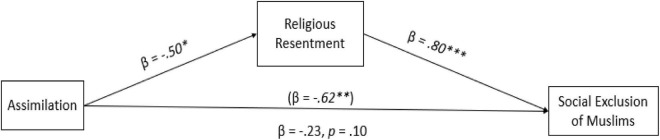
Path analysis between assimilation (2) vs the control (0) condition. The estimates in parentheses represent the direct effects before mediators were added to the model. Estimates are standardized. *p < 0.05. ^***^p < 0.001.

**FIGURE 5:**
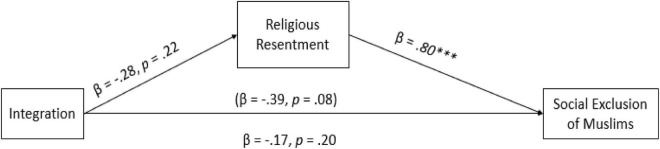
Path analysis between integration (1) vs the control (0) condition. The estimates in parentheses represent the direct effects before mediators were added to the model. Estimates are standardized. *p < 0.05. ^***^p < 0.001.

**FIGURE 6:**
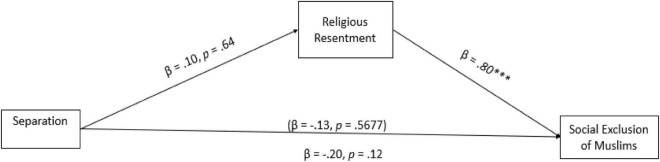
Path analysis between separation (3) vs the control (0) condition. The estimates in parentheses represent the direct effects before mediators were added to the model. Estimates are standardized. ^***^p < 0.001.

**FIGURE 7:**
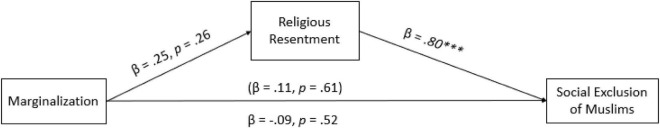
Path analysis between marginalization (4) vs the control (0) condition. The estimates in parentheses represent the direct effects before mediators were added to the model. Estimates are standardized *p < 0.05. ^***^p < 0.001.

As illustrated in Figure 4, the results showed that the assimilation condition indirectly decreased the social exclusion of Muslims due to low degrees of religious resentment toward Muslims (H3), indirect effect: B = -0.51, 95% CI [–0.96, –0.08]. The results imply that when Muslims were perceived as assimilated in the Canadian society, the participants indicated lower levels of religious resentment toward Muslims which decreased the social exclusion of Muslims from Canadian society. However, no significant indirect effects were found when religious resentment mediated the relationship between integration (Figure 5), indirect effect: B = -0.28, 95% CI [–0.74,0.17], separation, indirect effect: B = 0.08, 95% CI [–0.29,0.47] and marginalization conditions, indirect effect: B = 0.20, 95% CI [–0.16,0.55] and social exclusion (Figures 6, 7), partially confirming H3.

## Discussion

The main purpose of this experimental study was to examine whether religious resentment explains the social exclusion of Muslims from Canadian society as an outcome of Muslims’ acculturation strategies as perceived by majority group members using a vignette-based approach. The study revealed one area of great importance.

The investigated mediated relationships were only significant in the assimilation condition. Unlike the U.S, which is known as a “melting pot”, Canada is viewed as a multicultural “mosaic”, and a “country of immigrants”, where minorities are encouraged to maintain their heritage and religious customs and traditions in addition to adherence to the majority society’s culture (Litchmore and Safdar, 2015). Research suggests that minorities are viewed more favorably by majority group members when they indicate a preference for either assimilation or integration (Matera et al., 2011), while integration strategy is observed as a predictor of social inclusion of minorities (Zagefka and Brown, 2002). In the present study, we expected that Muslims’ social exclusion by majority group members would decrease when they were viewed as integrated or assimilated in Canadian society, while social exclusion would increase when they were viewed as separated and/or as a marginalized community. However, we found partial support for the former expectations, while we found no support for the latter ones. The relationship between Muslims’ perceived acculturation strategies and social exclusion was only significant when Muslims were presented as assimilated into society. No other relationship was significant with respect to other acculturation categories.

In the present study, the low means on social exclusion of Muslims, and religious resentment scales (see Table 2) reported by the participants indicate a weak tendency toward these attitudes in our sample. Therefore, we argue that even though the results showed a significant negative effect on the social exclusion of Muslims only in the assimilation condition, the results do not imply that Canadian society promotes the social exclusion of Muslim minorities. The main conclusion that emerged from this study, which is consistent with previous research, is that the majority group members in our study showed a preference for Muslims when they were perceived to adopt an assimilationist acculturation strategy (Bloom et al., 2015). One explanation of the result is that since assimilation implies low cultural dissimilarity with respect to language, values, and beliefs, majority group members are not only positively inclined toward Muslims who they perceived as assimilated but are also more likely to form social relationships with such individuals (Kunst and Sam, 2014).

In addition, individuals tend to view their group membership positively indicating in-group favoritism (Tajfel, 1982). Hence, Muslims who were perceived as assimilated into the majority society may be socially categorized as members of the majority in-group by the participants. This positive in-group categorization may have resulted in a favorable attitude toward Muslims by the participants. Thus, a second explanation for the result is that the participants socially included Muslims who they perceived as a part of their in-group due to similar values and cultural habits (Osbeck et al., 1997; Piontkowski et al., 2000; Matera et al., 2020). The findings also confirm previous research, which states that intergroup relations between the majority and minority groups are dependent on whether their acculturation attitudes are in accord with each other. When the majority group members prefer the minorities to assimilate, and the minority group adopts the mainstream culture, a consensual acculturative outcome is achieved resulting in positive intergroup relations such as social inclusion of the minorities (Rohmann et al., 2008), as it is also evident in the present study. In addition, the participant’s preference for socially including Muslims that are perceived as assimilated into the Canadian society and showing low degrees of resentment toward them reflects the European trend. In the assimilation condition, Muslims were socially included because Muslims’ religious practices were replaced with Canadian cultural habits, which portrays Muslims as less threatening to national security while at the same time their adoption of Canadian cultural habits is viewed as more liberal and democratic by the participants, hence, lowering feelings of resentment toward Muslims’ religious behavior (Helbling and Traunmuller, 2020).

Previous research has shown that Muslims neither prefer to separate or assimilate into Canadian society, but instead prefer integration as a strategy to engage in Canadian society (Yousif, 1992). However, it is surprising that in the current study, the participants did not consider Muslims as integrated in the Canadian society as the relationship between integration and social exclusion was not significant. These results may again support the idea that majority group members prefer cultural similarity with minorities and are more socially favorable toward minorities when they prefer a common in-group identification (see for example, Kunst et al., 2019). In line with this reasoning, we argue that the social exclusion of Muslims was not significantly endorsed by the majority group members when Muslims were presented as integrated, because it implied maintaining their religious culture as well as the Canadian culture. The present study showed that for the participants, the similarity of cultural values is an important predictor of less social exclusion of Muslims. Therefore, the finding that only the assimilation condition was a salient predictor of decreased levels of social exclusion of Muslims in Canada, while integration, separation, and marginalization did not decrease or increase social exclusion of Muslims, as expected, requires further investigation.

## Limitations and future implications

Our study, even though it contributes to the acculturation literature, has some limitations. First, we must take into consideration that the study took place during the rapidly evolving COVID-19 pandemic. The data was collected when lockdowns were imposed globally and Canada, along with the rest of the world, closed its borders to contain the pandemic. The uncertain and unexpected nature of the circumstances might have affected the participants’ opinions on matters related to Muslims’ presence in Canada. Indeed, a study conducted during the COVID pandemic found that Canadians supported stricter immigration policies, and promoted closed borders (Newbold, 2020). In addition, shaped by the pandemic, anti-Muslim hate crimes where a man drove a car into a Muslim family in London, Ontario killing four out of five family members as well as demonstrations against Islam as a religion in Canada took place (Ellsworth, 2022). Thus, we suggest a follow-up study with the same sample characteristics to examine whether these effects are significant when the pandemic mitigates.

Second, we used Mturk to recruit participants for the study, which has the advantage of attaining an adequate sample size in a short period of time. However, a disadvantage is that the participants might have prior experience in taking surveys, which may cause response bias. Moreover, online experiments only include a certain segment of a society that has access to the internet. Hence, diverse opinions are excluded from this study. Therefore, we recommend future studies take a mix-method approach where they interview individuals who may not have access to or the competence for using the internet, such as the older generation. Additionally, the vignettes explaining various acculturation strategies preferred to either maintain or adopt religious and majority society culture could be confusing for the participants to understand. Therefore, in order to maintain the validity of the vignettes, we suggest future studies use simpler words and smaller texts to clearly represent the intended acculturation strategy when describing the acculturation profiles. Additionally, future studies may also use implicit attitude tests to assess whether Muslims’ perceived acculturation strategies would yield social exclusion from society.

Moreover, in the assimilation vignette “preference for beer” was used as an indicator of assimilation into Canadian society compared to other vignettes. This behavioral preference of Muslims in the assimilation condition indicates a strong rejection of Islamic cultural practice because refusing the consummation of alcohol indicates obedience and adherence to Islamic values (Bagasra and Mackinem, 2019). Thus, Muslims perceived as preferring to drink beer may have influenced participants’ perceptions of Muslims as rejecting Islamic cultural identity and adopting Canadian social values, thus showing significant results in the assimilation condition compared to other conditions.

Another potential limitation of the study might be that the current study did not include a manipulation check item. However, the experiment included an attention check item (What is the name of the sport in the text?). This was done to ensure that the participants read the text and do not rush through the assigned vignette, without paying attention to the wording of the text. Therefore, instead of a direct manipulation check, an attention check item also known as a trap question was included in the experiment (Oppenheimer et al., 2009).

The results of this study have practical implications. While Canadian multicultural policy at the state level promotes the maintenance of heritage, religious, and majority society’s culture, the results showed that the participants only favored Muslims who were perceived as assimilated into Canadian society. We recommend future studies investigate other factors that might explain why assimilation was the only significant predictor of social inclusion, while integration did not reveal any significant results. One suggestion is to present Muslims’ cultural practices as a threat to Canadian cultural identity to examine the underlying mechanism for preferring social inclusion for assimilated Muslims (see e.g., Tahir et al., 2019). Moreover, even though Canadian Muslims are integrated into Canadian society (McCoy et al., 2016), the perception is different among majority group members. Thus, we propose that Muslim religious leaders, social scientists, and subject persons encourage a widespread acceptance of Muslim religious practices, and shared values with the majority society. Furthermore, Muslim religious leaders and policymakers are recommended to promote Muslims’ religious and mainstream cultural association as an integral part of their Canadian Muslim identity so that Canada circumvents the European trend, where the idea of inclusivity of various cultural identities is being replaced by a single cultural identity based on the majority group’s culture (Ouseley, 2001). In this study, we found that religious resentment had an omnibus effect on the social exclusion of Muslims. This finding will be of interest to organizations working in the social inclusion domain. Specifically, policymakers, law enforcement agencies, and Muslim religious leaders are suggested to collaborate to create common grounds for intergroup and interfaith dialog and information exchange. An important policy can be to create social contexts that promote positive intergroup interactions in the effort to combat negative feelings toward Muslims who choose to integrate, instead of assimilation, or are on the verge of marginalization in their society of the living. This approach will also provide platforms to the majority society where they can learn positive information about Muslims and Islam (see e.g., Barise, 2005), which may reduce any gap that was created between the Muslim minority and the majority society because of past grievances.

## Data availability statement

The raw data supporting the conclusions of this article will be made available by the authors, without undue reservation.

## Ethics statement

The studies involving human participants were reviewed and approved by Research Ethics Board, University of Guelph Canada. The patients/participants provided their written informed consent to participate in this study. Written informed consent was obtained from the individual(s) for the publication of any potentially identifiable images or data included in this article.

## Author contributions

HT: conception and design, data collection and analysis, and drafting of the article. SS: interpretation of data, supervision of the data collection, and critical revision of the manuscript for important intellectual content. Both authors approved the final version to be published.
